# Association between handgrip strength and visceral obesity with low appendicular skeletal muscle mass in Chinese adults

**DOI:** 10.3389/fendo.2026.1857193

**Published:** 2026-06-24

**Authors:** Dingliu He, Tong Zhao, Guanhao Qiao, Xiaochen Shen, Shuai Lu, Yuanyuan Wang, Jianxia Wu, Dingming Sun, Chunxia Ge

**Affiliations:** 1 Center of Health Management, Yancheng Clinical College of Xuzhou Medical University, The First People’s Hospital of Yancheng, Yancheng, China; 2 Department of Clinical Nutrition, Yancheng Clinical College of Xuzhou Medical University, The First People’s Hospital of Yancheng, Yancheng, China; 3 Department of Clinical Nutrition, Yancheng Third People’s Hospital, Affiliated Hospital 6 of Nantong University, Yancheng, Yancheng, China

**Keywords:** appendicular skeletal muscle mass, co-occurrence, handgrip strength, obesity, visceral obesity

## Abstract

**Objective:**

Although handgrip strength (HGS) has been shown to be associated with various health outcomes, its relationship with visceral obesity and low appendicular skeletal muscle mass (ASM) has rarely been examined. This study aimed to investigate the independent associations between HGS and the risk of visceral obesity, low ASM, and their co-occurrence in the Chinese population.

**Methods:**

The data we used were obtained from individuals who attended the health checkup program at the health management center of Yancheng First People’s Hospital from March 2023 to November 2024. The odds ratio (OR) and 95% confidence interval (CI) for visceral obesity, low ASM, and their co-occurrence were calculated using a logistic regression model with the lowest HGS group serving as the reference category.

**Results:**

After adjusting for BMI, age, sex and other covariates, subjects in the highest quartile of HGS were significantly associated with a lower risk of visceral obesity (OR: 0.14, 95% CI: 0.08-0.27, *p* < 0.001), low ASM (OR: 0.10, 95% CI: 0.05-0.18, *p* < 0.001), and their co-occurrence (OR: 0.09, 95% CI: 0.04-0.22, *p* < 0.001). For visceral obesity, HGS showed an L-shaped, nonlinear association in females, characterized by a sharp reduction in risk at lower HGS levels, followed by a plateau at higher HGS levels (*p* for nonlinearity < 0.05). Meanwhile, the area under the curve (AUC) values for low ASM, visceral obesity, and their co-occurrence were 0.88 (0.86, 0.89), 0.93 (0.92, 0.94), and 0.72 (0.67, 0.76), respectively.

**Conclusion:**

Higher HGS was associated with a lower risk of visceral obesity, low ASM, and their co-occurrence.

## Introduction

Obesity has emerged as an intractable worldwide health concern in recent years. In China, the prevalence of overweight and obesity has increased from 29.9% in 2002 to 50.7% in 2018 (1). Additionally, the abdominal obesity rate of Chinese adults has also increased markedly in the past two decades (2). Excess adipose tissue, especially in the form of visceral obesity, has been identified as a major risk factor for cardiovascular disease, metabolic diseases and cancer (3, 4). These conditions represent substantial health and economic burdens (5, 6). Therefore, the assessment of visceral obesity is of utmost importance and is typically conducted using specialized medical equipment. However, this approach can be both inconvenient and costly for large-scale community screenings.

Previous studies have often evaluated obesity through body indices such as Body Mass Index (BMI) and waist circumference (WC) (7, 8). Although these indices are valuable for evaluating overall obesity, they are typically confined to a singular dimension and fall short in accurately reflecting visceral obesity, particularly when combined with reduced appendicular skeletal muscle mass (ASM) in individuals.

Handgrip strength (HGS) serves as a crucial indicator of muscular strength and plays an important role in identifying the risk of sarcopenia (9). Meanwhile, multiple studies reported that low adjusted HGS was significantly associated with abdominal obesity (10, 11). However, there has been limited research focusing on the association between HGS and visceral obesity. Therefore, our research aims to present the associations of HGS with visceral obesity, low ASM, and their co-occurrence among Chinese adults.

## Methods

### Study population

**Figure 1:**
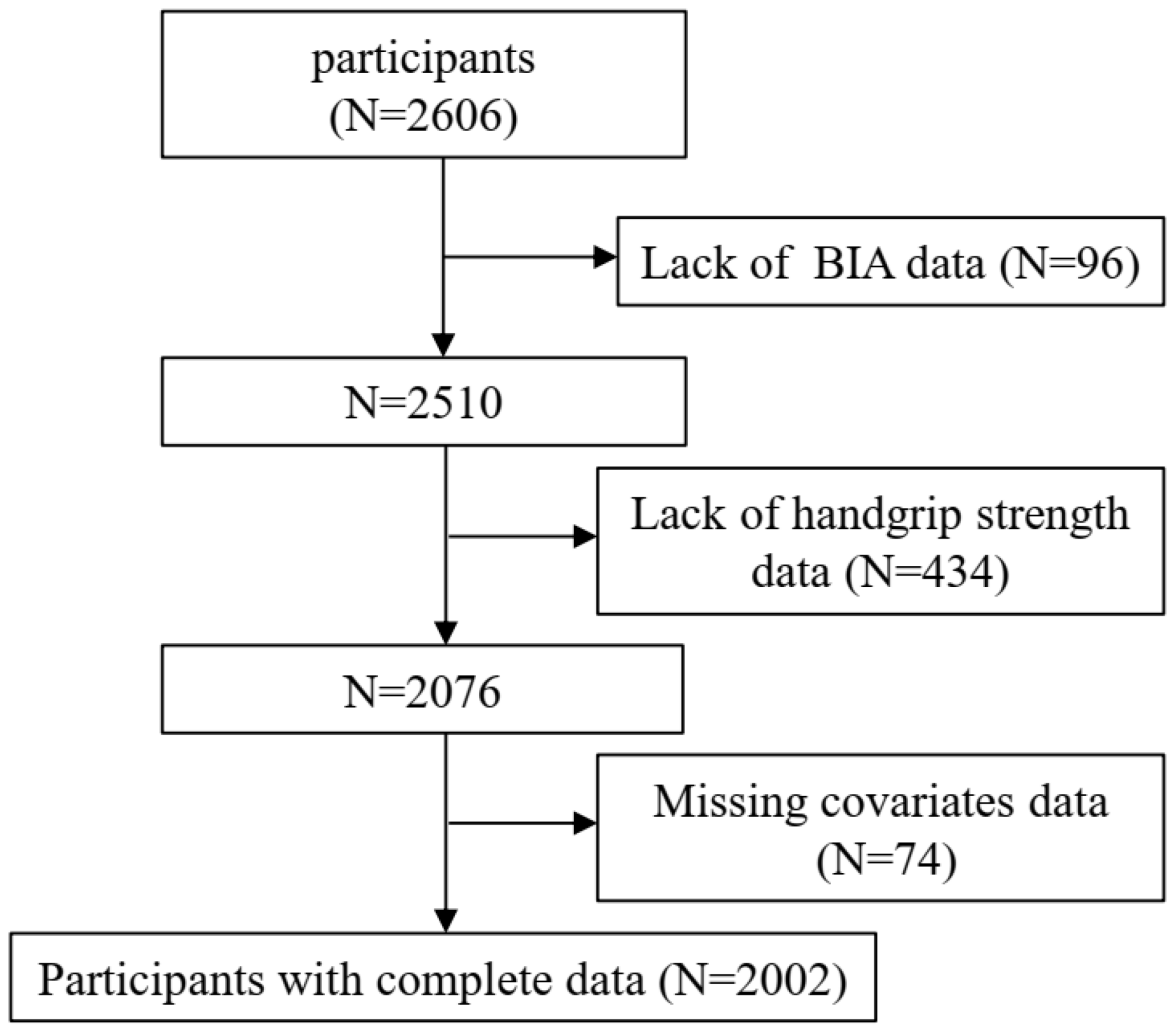
The flow chart of the sample selection methods in each step.

The data we used were obtained from individuals who attended the health checkup program at the health management center of Yancheng First People’s Hospital from March 2023 to November 2024. After excluding participants under the age of 18, pregnant women, and those unable to complete physical measurements, a total of 2002 individuals were ultimately included in the analysis. The flow chart of the sample selection process is shown in **Figure 1**. All methods were performed in accordance with the ethical principles of the Declaration of Helsinki, and all participants signed informed consent forms. This study was approved by the ethics review committee of Yancheng First People’s Hospital (Approval Number:2023K233).

### Assessment of HGS, visceral obesity and low ASM

HGS: We use the electronic grip strength meter (CAMRY, EH101, China) to measure HGS. The doctor asked the participants which hand was their dominant hand. If the response is left or right, measure the grip strength of that hand. If the response is “I don’t know” or “about the same”, measure the grip strength of both hands. The subject stands with their feet parallel and slightly spread to shoulder width, with their arms naturally hanging to their sides. Then, using their dominant hand, grip the device tightly and keep holding it for 3 seconds. The doctor ensures that the reading of the device is reset to zero before the measurement begins. Three measurements were obtained for each hand, and the highest value was recorded (12). The bioelectrical impedance analysis (BIA, Inbody 720, Korea) was used to measure the visceral fat area and ASM. For the evaluation of BIA, height was measured by a researcher using the Omron^®^ device (SK-L108, China), which has a resolution of 1 mm. During the assessment, participants maintained an orthostatic position while holding two levers and positioning their feet on a platform. The evaluation lasted approximately 3 minutes. All subjects were instructed to adhere to specific recommendations, which included fasting for at least 8 hours, wearing light clothing, being barefoot, avoiding any metal objects, and ensuring they had already urinated and defecated before the assessment. Evaluations were conducted in the morning between 7 a.m. and 10 a.m. A visceral fat area of ≥100 cm^2^ was defined as visceral obesity (13). Height-adjusted ASM was calculated as ASM/height^2^ (kg/m^2^). The low ASM was defined as follows: for males, when age<65 years and height-adjusted ASM < 7.6 kg/m^2^, or age ≥ 65 years and height-adjusted ASM<7.0 kg/m^2^; for females, height-adjusted ASM<5.7 kg/m^2^ (9). The co-occurrence was defined as the combination of visceral obesity and low ASM. Specifically, participants who met the criteria for both conditions were classified as having the co-occurrence. In our study, HGS was used as the primary exposure variable and three outcomes were defined, including visceral obesity, low ASM and their co-occurrence.

### Other variables

In this study, education level was categorized into three tiers: middle school or below, high school, and college or above. Hypertension was defined as a systolic blood pressure (SBP) of ≥140 mmHg, a diastolic blood pressure (DBP) of ≥90 mmHg, self-reported hypertension, or the use of antihypertensive medication. Physical activity is categorized into two levels according to whether it is performed at least once per week (Yes/No). BMI (kg/m^2^) was calculated as weight divided by height squared. All the participants were kept in a standing position and breathed steadily. And then, WC was recorded at the end of exhalation by using the tape measure circle, based horizontally on the navel. The fasting blood sample from each participant was collected by a trained nurse. Blood samples were verified and analyzed in the Center for Clinical Laboratory of Yancheng First People’s Hospital.

### Statistical analysis

Data analysis was performed using the statistical software packages SAS 9.4 and R 4.5.2. Continuous variables were expressed as mean ± standard deviation (SD), while categorical variables were presented as frequency (percentage). A chi-square test was employed to identify statistical differences in sex, location, education level, smoking status, and alcohol consumption. The odds ratio (OR) and 95% confidence interval (CI) for outcomes were calculated using a logistic regression model with the lowest HGS group serving as the reference category. Restricted cubic spline (RCS) regression models were utilized to explore potential linear or non-linear relationships between continuous variables and outcomes; The performance of HGS for identifying each outcome was assessed through the area under the receiver operating characteristic (ROC) curve (AUC). The significance level was set at 0.05 (two-sided).

## Result

### Characteristics of the study cohort

The baseline characteristics of the subjects according to the quartiles of HGS are shown in **Table 1**. Our study consisted of 2002 individuals, including 1163 men and 839 women. The mean age of participants was 50.9 (14.8). Compared with those in the lower quartiles, participants in higher HGS quartiles were likely to be male, drinkers and smokers. They also presented higher levels of BMI, WC, triglycerides, visceral fat area, glucose, SBP and DBP, alongside lower concentrations of high-density lipoprotein cholesterol (HDL-C).

**Table 1:** Baseline characteristics of participants classified by quartiles of HGS.

Characteristics	All	Q1 (<27.2)	Q2 (27.2-34.8)	Q3 (34.8-42.6)	Q4 (≥42.7)	*P* value
N	2002	506	501	499	496	
Male (n, %)	1163 (58.1)	30 (5.9)	166 (33.1)	476 (95.4)	491 (98.9)	<0.001
Age (years)	50.9 ± 14.8	54.4 ± 15.3	53.1 ± 15.0	51.0 ± 13.9	45.0 ± 13.0	<0.001
BMI (kg/m^2^)	24.6 ± 3.8	23.0 ± 3.2	24.0 ± 3.3	25.3 ± 3.2	26.2 ± 4.4	<0.001
WC (cm)	83.4 ± 11.1	76.2 ± 9.2	79.8 ± 10.8	87.9 ± 8.8	89.8 ± 9.4	<0.001
Education level						<0.001
middle school or below	256 (12.8)	81 (16.0)	83 (16.6)	52 (10.4)	40 (8.1)	
high school	278 (13.9)	81 (16.0)	64 (12.8)	79 (15.8)	54 (10.9)	
college or above	1468 (73.3)	344 (68.0)	354 (70.6)	368 (73.8)	402 (81.0)	
Physical activity	1327 (66.3)	331 (65.4)	339 (67.7)	332 (66.5)	325 (65.5)	0.864
Visceral Fat Area (cm^2^)	98.0 ± 36.2	102.1 ± 37.8	100.0 ± 35.8	94.7 ± 34.6	95.1 ± 35.9	0.002
smoked	613 (30.6)	17 (3.4)	104 (20.8)	236 (47.3)	256 (51.6)	<0.001
Frequent drinking	543 (27.1)	18 (3.6)	70 (14.0)	208 (41.7)	247 (49.8)	<0.001
DBP (mmHg)	80.0 ± 12.2	76.3 ± 11.9	77.6 ± 11.6	83.4 ± 12.2	82.7 ± 11.4	<0.001
SBP (mmHg)	127.8 ± 19.0	125.1 ± 21.1	126.6 ± 20.3	130.9 ± 18.2	128.4 ± 15.6	<0.001
Hypertension	940 (47.0)	239 (47.2)	230 (45.9)	263 (52.7)	208 (41.9)	0.008
Glucose (mmol/l)	5.3 ± 1.3	5.0 ± 0.9	5.2 ± 1.2	5.5 ± 1.5	5.5 ± 1.5	<0.001
Triglycerides (mmol/l)	1.65 ± 1.39	1.38 ± 0.81	1.41 ± 0.81	1.86 ± 1.63	1.97 ± 1.88	<0.001
HDL-C (mmol/l)	1.41 ± 0.30	1.55 ± 0.32	1.48 ± 0.31	1.31 ± 0.25	1.32 ± 0.26	<0.001
LDL-C (mmol/l)	3.17 ± 0.72	3.19 ± 0.74	3.12 ± 0.69	3.18 ± 0.76	3.19 ± 0.68	0.3899

### The risk of visceral obesity with low ASM by the quartiles of HGS

OR (95% CI) for the association between quartiles of HGS and visceral obesity with low ASM are shown in **Table 2**. Compared with participants with the lowest HGS, participants in the highest quartile of HGS were at a lower risk of visceral obesity (OR: 0.64, 95% CI: 0.50-0.82, *p* = 0.001). After further adjusting for a series of confounding factors, the OR (95% CI) was attenuated to 0.14 (0.08, 0.27) for participants with the highest HGS. Meanwhile, participants in the highest quartile of HGS also had a lower risk of low ASM (OR: 0.10, 95% CI: 0.05-0.18, *p* < 0.001). Furthermore, those in the higher quartile of HGS have a lower risk of visceral obesity when combined with low ASM (OR: 0.09, 95% CI: 0.04-0.22, *p* < 0.001). These results are robust in the sex stratified analysis, which showed that the inverse associations were present in both sexes but were stronger in females (**Tables 3**, **4**). In the sensitivity analysis without BMI adjustment (**Supplementary Table 1**), the inverse associations between HGS and visceral obesity were no longer significant, and other results remained robust.

**Table 2:** OR (95% CI) of the association between HGS and visceral obesity with low ASM in Chinese adults.

Outcomes	OR (95% CI)					
	Model 1	*P*	Model 2	*P*	Model 3	*P*
Visceral obesity						
Q1	1.00 (Ref)		1.00 (Ref)		1.00 (Ref)	
Q2	0.88 (0.69, 1.13)	0.321	0.54 (0.37, 0.80)	0.002	0.54 (0.37, 0.79)	0.002
Q3	0.65 (0.50, 0.83)	0.001	0.28 (0.16, 0.51)	<0.001	0.27 (0.15, 0.49)	<0.001
Q4	0.64 (0.50, 0.82)	0.001	0.15 (0.08, 0.28)	<0.001	0.14 (0.08, 0.27)	<0.001
Low ASM						
Q1	1.00 (Ref)		1.00 (Ref)		1.00 (Ref)	
Q2	0.61 (0.47, 0.80)	<0.001	0.31 (0.21, 0.45)	<0.001	0.30 (0.20, 0.44)	<0.001
Q3	1.07 (0.83, 1.38)	0.620	0.25 (0.15, 0.44)	<0.001	0.24 (0.13, 0.42)	<0.001
Q4	0.48 (0.36, 0.63)	<0.001	0.11 (0.06, 0.19)	<0.001	0.10 (0.05, 0.18)	<0.001
Visceral obesity and Low ASM						
Q1	1.00 (Ref)		1.00 (Ref)		1.00 (Ref)	
Q2	0.45 (0.28, 0.72)	<0.001	0.30 (0.17, 0.52)	<0.001	0.30 (0.18, 0.52)	<0.001
Q3	0.61 (0.39, 0.93)	0.001	0.24 (0.12, 0.48)	<0.001	0.23 (0.11, 0.47)	<0.001
Q4	0.25 (0.14, 0.45)	0.023	0.10 (0.04, 0.22)	<0.001	0.09 (0.04, 0.22)	<0.001

Model 1 adjusted for no variable. Model 2 adjusted for BMI, age, sex and education. Model 3 adjusted for BMI, age, sex, education, smoked, alcohol drinking, glucose, high-density lipoprotein cholesterol (HDL-C), low-density lipoprotein cholesterol (LDL-C), triglycerides, SBP and DBP.

**Table 3:** OR (95% CI) of the association between HGS and visceral obesity with low ASM in Chinese male adults.

Outcomes	OR (95% CI)					
	Model 1	*P*	Model 2	*P*	Model 3	*P*
Visceral obesity						
Q1	1.00 (Ref)		1.00 (Ref)		1.00 (Ref)	
Q2	0.89 (0.64, 1.24)	0.494	0.48 (0.29, 0.79)	0.004	0.45 (0.27, 0.74)	0.002
Q3	0.71 (0.51, 1.00)	0.053	0.32 (0.19, 0.53)	<0.001	0.30 (0.18, 0.51)	<0.001
Q4	1.01 (0.73, 1.41)	0.946	0.25 (0.14, 0.43)	<0.001	0.23 (0.13, 0.41)	<0.001
Low ASM						
Q1	1.00 (Ref)		1.00 (Ref)		1.00 (Ref)	
Q2	0.62 (0.45, 0.87)	0.005	0.64 (0.42, 0.98)	0.040	0.64 (0.45, 0.90)	0.011
Q3	0.41 (0.29, 0.58)	<0.001	0.33 (0.21, 0.52)	<0.001	0.37 (0.26, 0.53)	<0.001
Q4	0.19 (0.13, 0.27)	<0.001	0.18 (0.11, 0.30)	<0.001	0.17 (0.12, 0.26)	<0.001
Visceral obesity and Low ASM						
Q1	1.00 (Ref)		1.00 (Ref)		1.00 (Ref)	
Q2	0.43 (0.23, 0.78)	0.005	0.36 (0.19, 0.67)	0.001	0.35 (0.19, 0.66)	0.001
Q3	0.35 (0.18, 0.65)	0.001	0.28 (0.14, 0.55)	<0.001	0.25 (0.13, 0.50)	<0.001
Q4	0.22 (0.10, 0.47)	<0.001	0.16 (0.07, 0.37)	<0.001	0.16 (0.07, 0.36)	<0.001

Model 1 adjusted for no variable. Model 2 adjusted for BMI, age and education. Model 3 adjusted for BMI, age, education, smoked, alcohol drinking, glucose, HDL, LDL, triglycerides, SBP and DBP.

**Table 4:** OR (95% CI) of the association between HGS and visceral obesity with low ASM in Chinese female adults.

Outcomes	OR (95% CI)					
	Model 1	*P*	Model 2	*P*	Model 3	*P*
Visceral obesity						
Q1	1.00 (Ref)		1.00 (Ref)		1.00 (Ref)	
Q2	0.79 (0.54, 1.17)	0.238	0.74 (0.42, 1.33)	0.315	0.69 (0.38, 1.25)	0.217
Q3	0.97 (0.66, 1.43)	0.885	0.52 (0.29, 0.93)	0.028	0.48 (0.26, 0.87)	0.015
Q4	0.81 (0.56, 1.19)	0.287	0.28 (0.16, 0.52)	<0.001	0.26 (0.14, 0.49)	<0.001
Low ASM						
Q1	1.00 (Ref)		1.00 (Ref)		1.00 (Ref)	
Q2	0.59 (0.40, 0.88)	0.010	0.42 (0.25, 0.70)	0.001	0.39 (0.23, 0.67)	<0.001
Q3	0.29 (0.19, 0.45)	<0.001	0.25 (0.14, 0.43)	<0.001	0.23 (0.13, 0.41)	<0.001
Q4	0.10 (0.05, 0.18)	<0.001	0.10 (0.05, 0.18)	<0.001	0.09 (0.04, 0.18)	<0.001
Visceral obesity and Low ASM						
Q1	1.00 (Ref)		1.00 (Ref)		1.00 (Ref)	
Q2	0.44 (0.24, 0.80)	0.008	0.50 (0.27, 0.92)	0.027	0.46 (0.25, 0.88)	0.018
Q3	0.22 (0.10, 0.47)	<0.001	0.27 (0.12, 0.58)	0.001	0.24 (0.11, 0.53)	<0.001
Q4	0.02 (0.01, 0.17)	<0.001	0.03 (0.01, 0.24)	0.001	0.03 (0.01, 0.22)	0.001

Model 1 adjusted for no variable. Model 2 adjusted for BMI, age and education. Model 3 adjusted for BMI, age, education, smoked, alcohol drinking, glucose, HDL, LDL, triglycerides, SBP and DBP.

### The relationship between HGS and various outcomes by RCS

**Figure 2:**
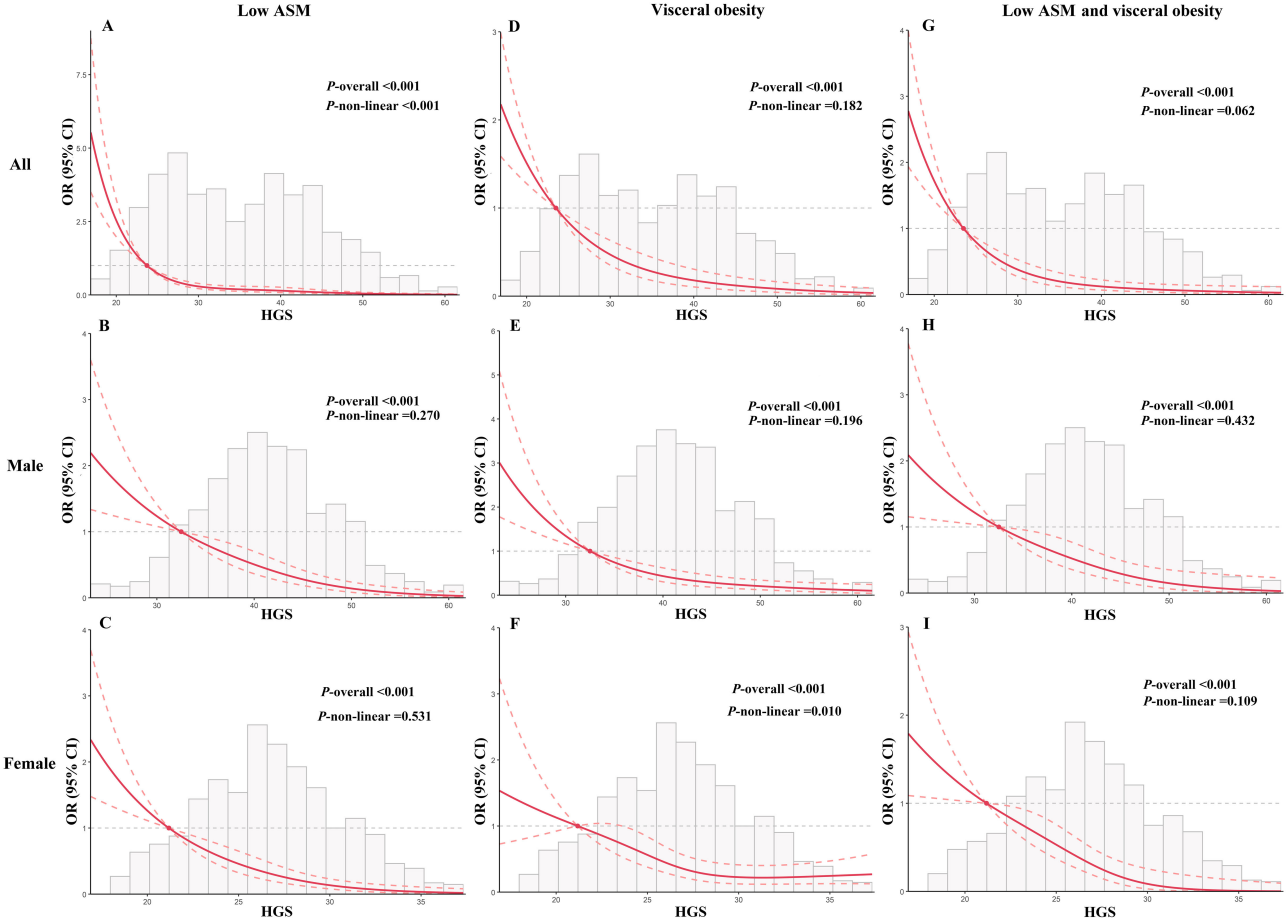
Restricted cubic spline curves for analyzing the relationship between HGS and low ASM **(A–C)**, visceral obesity **(D–F)**, and their co-occurrence **(G–I)**.

The RCS analyses further revealed a dose-response relationship between HGS and various outcomes (**Figures 2A–I**). For visceral obesity, HGS exhibited an L-shaped nonlinear association in females, characterized by a sharp reduction in risk at lower HGS levels, followed by a plateau at higher HGS levels (*p* for nonlinearity < 0.05; **Figure 2F**). For low ASM, the HGS also exhibited an L-shaped and nonlinear association across all participants (*p* values for nonlinearity<0.05; **Figure 2A**).

### The AUC values of HGS for identifying different outcomes

**Figure 3:**
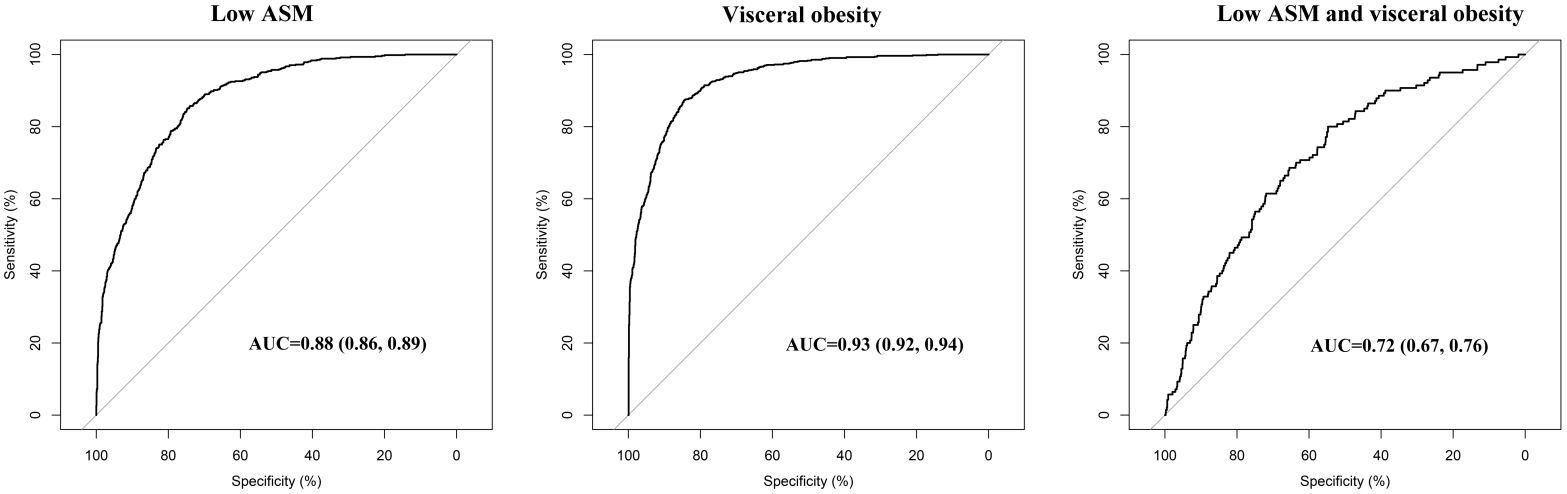
Receiver operating characteristic curve analysis of HGS for identifying visceral obesity, low ASM, and their co-occurrence.

**Figure 3** shows the AUC values of HGS for identifying low ASM, visceral obesity, and their co-occurrence. The AUC values for low ASM, visceral obesity, and their co-occurrence were 0.88 (0.86, 0.89), 0.93 (0.92, 0.94), and 0.72 (0.67, 0.76), respectively.

## Discussion

Based on a sample of 2002 adults in China, we comprehensively evaluated the associations of HGS with visceral obesity, low ASM, and their co-occurrence in individuals. Our research found that higher HGS was associated with a lower risk of visceral obesity, low ASM, as well as with their co-occurrence. These associations remained robust in sex-stratified analyses, revealing stronger protective effects in females than in males. Meanwhile, a nonlinear association was observed between HGS and visceral obesity among women. Furthermore, HGS demonstrated high AUC values for identifying visceral obesity, low ASM, and their co-occurrence.

Currently, a large body of evidence has confirmed that HGS can effectively predict a variety of health conditions, making it a valuable biomarker for assessing overall physiological reserve and the aging process (14–16). One systematic review incorporating eight meta-analyses reported that higher handgrip strength (HGS) was significantly associated with nine health outcomes, such as lower risks of all-cause mortality, cardiovascular mortality, and disability (17). In line with the existing literature, our findings confirm that higher HGS was significantly associated with a lower risk of visceral obesity. Although the focus of obesity research has shifted from general obesity to visceral fat (18), studies directly examining the correlation between grip strength and visceral fat remain relatively scarce. Notably, one study in a large Chinese cohort of 15,820 adults reported that relative HGS was inversely associated with metabolic syndrome, which is closely linked to visceral adiposity, and this association was stronger in women than in men, which is consistent with our sex-specific findings (19). Unlike previous studies that mainly focused on general obesity assessed by BMI and WC, our study specifically focused on visceral obesity, the region where metabolically active fat accumulates. Meanwhile, a notable finding of our study was a nonlinear association between HGS and visceral obesity, observed only in women. From a clinical perspective, this nonlinearity implies that interventions to increase muscle strength may yield the greatest metabolic benefits in individuals with low or moderate baseline strength. In our study, the sensitivity analysis that removed BMI from the fully adjusted model showed that the inverse association between HGS and visceral obesity became non-significant. Nevertheless, extensive evidence from causal inference frameworks supports that adjusting for BMI is necessary to avoid negative confounding (20, 21). This pattern can be explained by the fact that higher BMI increases both HGS and visceral fat, thereby masking the true effect of HGS.

The inverse association between HGS and low ASM was robust across all analyses. These findings are consistent with previous studies. In a cross-sectional study of 1,748 Chinese adults, the prevalence of low ASM increased from 21.5% in the normal HGS group to 39.5% in the low HGS group, and low HGS was independently associated with low muscle mass (OR = 1.7, 95% CI:1.203-2.402) (22), consistent with our findings. To date, the European Working Group on Sarcopenia in Older People (EWGSOP) and the Asian Working Group for Sarcopenia (AWGS) have both incorporated HGS as a key diagnostic criterion for sarcopenia (9, 23). Moreover, the strongest inverse association in our study was observed between HGS and the concurrent presence of visceral obesity and low ASM. This condition reflects a dual metabolic burden, wherein excess adiposity and inadequate skeletal muscle mass act synergistically to increase the risk of disability, metabolic disorders, and premature mortality (24, 25). Similarly, we observed sex differences, with higher HGS conferring stronger protective effects in women. This may be explained by distinct fat distribution patterns, where men predominantly accumulate visceral fat and women tend to accumulate subcutaneous fat, which is metabolically less deleterious (26). Moreover, estrogen exerts protective effects on skeletal muscle by enhancing protein synthesis and reducing inflammation (27).

In addition, HGS demonstrated high AUC values for identifying visceral obesity, low ASM, and their co-occurrence. In contrast, conventional measures such as BMI and WC have significantly lower discriminatory power for obesity related outcomes (the AUC values consistently<0.70) (28). Moreover, they cannot distinguish visceral from subcutaneous fat nor capture muscle loss (29). However, the high ROC for HGS in identifying low ASM may partly be attributed to their inherent relationship rather than representing an independent discriminatory capacity. Nonetheless, further longitudinal cohort research is needed to determine the optimal discriminatory parameters for clinical applications.

The potential mechanisms linking HGS to visceral obesity with low ASM could be attributed to several factors. First, individuals with higher HGS generally exhibit better insulin sensitivity (30, 31), which can directly inhibit the accumulation of visceral fat and promote the synthesis of muscle proteins (32, 33). Second, previous research shows that lower skeletal muscle strength was significantly associated with higher levels of circulating inflammatory markers, such as C-reactive protein, Interleukin-6, and Tumor necrosis factor (34). However, higher levels of inflammation in individuals can significantly increase the risk of adipose tissue accumulation and muscle mass loss (35). Third, lower HGS is often associated with reduced physical activity and exercise levels (36). These activities can independently prevent the accumulation of visceral fat by increasing energy expenditure and promoting fat breakdown, while also promoting muscle protein synthesis (37, 38). These mechanisms may explain how HGS is associated with visceral fat and ASM.

The strengths of this study include the large sample size of participants in the real world. The inclusion of 2,002 Chinese adults provides sufficient statistical power for stratified analyses. Additionally, we clearly show that HGS was associated with a lower risk of visceral obesity and low ASM, as well as with their combined condition. However, limitations also exist in this study. First, our research was a cross-sectional design, which naturally limits the inference of causal relationships. Longitudinal studies are needed to establish temporal relationships and directionality. Second, the study sample comprised Chinese adults recruited from a single health management center, a population that is generally healthier, more educated, and socioeconomically advantaged; thus, our findings may not be generalizable to the broader Chinese population or to other populations with different demographic characteristics. Third, visceral fat was assessed using BIA, whereas CT and MRI are regarded as the gold standard methods for quantifying visceral adipose tissue. However, recent studies have shown that the BIA method is effective in measuring visceral fat (39). Fourth, we did not exclude participants with prior cerebrovascular disease, musculoskeletal disorders, or other conditions, which may have introduced residual confounding in HGS measurement and thereby potentially affected the exploration of the associations.

In conclusion, this study demonstrates that higher handgrip strength is significantly associated with lower risks of visceral obesity, low ASM, and their co-occurrence in Chinese adults. These findings support further investigation of HGS as an accessible and inexpensive marker of metabolic and musculoskeletal health.

## Data Availability

The original contributions presented in the study are included in the article/**Supplementary Material**. Further inquiries can be directed to the corresponding author.
