# Supplementary material for: Association between handgrip strength and visceral obesity with low appendicular skeletal muscle mass in Chinese adults
Source: Front Endocrinol (Lausanne). 2026 Jun 24;17:1857193. doi: 10.3389/fendo.2026.1857193 (PMC13341461; doi:10.3389/fendo.2026.1857193)
Supplement: Supplementary file 1 [file Table1.docx]

**Association between handgrip strength and visceral obesity with low appendicular skeletal muscle mass in Chinese adults**

Supplement Table 1. OR (95% CI) of the association between HGS and visceral obesity with low ASM in Chinese adults

| Outcomes | OR (95% CI) | |
| --- | --- | --- |
|  | Model | *P* |
| Visceral obesity |  |  |
| Q1 | 1.00 (Ref) |  |
| Q2 | 0.99 (0.75, 1.31) | 0.967 |
| Q3 | 0.85 (0.57, 1.29) | 0.451 |
| Q4 | 0.91 (0.59, 1.41) | 0.676 |
| Low ASM |  |  |
| Q1 | 1.00 (Ref) |  |
| Q2 | 0.30 (0.22, 0.43) | <0.001 |
| Q3 | 0.20 (0.13, 0.32) | <0.001 |
| Q4 | 0.08 (0.05, 0.13) | <0.001 |
| Visceral obesity and Low ASM |  |  |
| Q1 | 1.00 (Ref) |  |
| Q2 | 0.31 (0.18, 0.53) | <0.001 |
| Q3 | 0.24 (0.12, 0.48) | <0.001 |
| Q4 | 0.10 (0.04, 0.22) | <0.001 |

Model adjusted for age, sex, education, smoked, alcohol drinking, glucose, HDL, LDL, TG, SBP and DBP.
